# The Banana Transcriptional Repressor MaDEAR1 Negatively Regulates Cell Wall-Modifying Genes Involved in Fruit Ripening

**DOI:** 10.3389/fpls.2016.01021

**Published:** 2016-07-11

**Authors:** Zhong-qi Fan, Jian-fei Kuang, Chang-chun Fu, Wei Shan, Yan-chao Han, Yun-yi Xiao, Yu-jie Ye, Wang-jin Lu, Prakash Lakshmanan, Xue-wu Duan, Jian-ye Chen

**Affiliations:** ^1^State Key Laboratory for Conservation and Utilization of Subtropical Agro-bioresources/Guangdong Key Laboratory for Postharvest Science, College of Horticultural Science, South China Agricultural UniversityGuangzhou, China; ^2^Sugar Research Australia, BrisbaneQLD, Australia; ^3^Key Laboratory of Plant Resources Conservation and Sustainable Utilization, South China Botanical Garden, Chinese Academy of SciencesGuangzhou, China

**Keywords:** banana (*Musa acuminata*), cell wall modification, DREB, fruit ripening, transcriptional repression

## Abstract

Ethylene plays an essential role in many biological processes including fruit ripening via modulation of ethylene signaling pathway. Ethylene Response Factors (ERFs) are key transcription factors (TFs) involved in ethylene perception and are divided into AP2, RAV, ERF, and DREB sub-families. Although a number of studies have implicated the involvement of DREB sub-family genes in stress responses, little is known about their roles in fruit ripening. In this study, we identified a DREB TF with a EAR motif, designated as MaDEAR1, which is a nucleus-localized transcriptional repressor. Expression analysis indicated that *MaDEAR1* expression was repressed by ethylene, with reduced levels of histone H3 and H4 acetylation at its regulatory regions during fruit ripening. In addition, *MaDEAR1* promoter activity was also suppressed in response to ethylene treatment. More importantly, MaDEAR1 directly binds to the DRE/CRT motifs in promoters of several cell wall-modifying genes including *MaEXP1/3*, *MaPG1*, *MaXTH10*, *MaPL3*, and *MaPME3* associated with fruit softening during ripening and represses their activities. These data suggest that MaDEAR1 acts as a transcriptional repressor of cell wall-modifying genes, and may be negatively involved in ethylene-mediated ripening of banana fruit. Our findings provide new insights into the involvement of DREB TFs in the regulation of fruit ripening.

## Introduction

The phytohormone ethylene plays an essential role in many biological processes of plant growth and development, including germination, organ senescence, stress response, and fruit ripening (Salehin and Estelle, 2015). The ethylene signaling pathway is well studied in *Arabidopsis*, which reveals a linear transduction pathway with the transduction of ethylene signal from receptors to dedicated transcription factors (TFs) (Pirrello et al., 2012). The last components of the ethylene signaling pathway are the Ethylene Response Factor (ERF) TFs which possess a highly conserved DNA-binding domain called the APETALA2/ethylene-responsive element binding (AP2/ERF) domain (Ohme-Takagi and Shinshi, 1995).

The AP2/ERF proteins are divided into four major sub-families, namely the AP2, related to ABI3/VP1 (RAV), ERF and dehydration-responsive element-binding protein (DREB), according to the number and similarity of the AP2/ERF domains (Sakuma et al., 2002). DREB TFs, as a sub-family of the AP2/ERF proteins, were first isolated using an 6-bp conserved sequence (A/GCCGAC), named the dehydration responsive element (DRE), in yeast one-hybrid screening in *Arabidopsis* cDNA (Stockinger et al., 1997; Liu et al., 1998). Extensive studies have established important regulatory roles for DREB TFs in response to environmental stimuli. For example, in *Arabidopsis*, *DREB1A* was induced by cold, while *DREB2* like genes (*DREB2A* and *DREB2B*) were induced by drought, salt and heat (Nakashima et al., 2000). By contrast, other *DREB1*-related genes such as *DREB1D* regulate high osmotic stress-induced gene expression (Haake et al., 2002), whereas *DREB1E* and *DREB1F* are responsive to high salinity (Mizoi et al., 2012). Except for these transcriptional activators, several members of DREB TFs with ERF-associated amphiphilic repression (EAR) motif at C-terminus act as transcriptional repressors of stress responses (Ohta et al., 2001; Kagale and Rozwadowski, 2010). These EAR motif-containing DREB repressors negatively modulate the responses of plants to cold and dehydration, as are the cases of DEAR1 (Tsutsui et al., 2009), RAP2.1 (Dong and Liu, 2010), and GhDREB (Gao et al., 2009). Despite these findings, less is known about the functions of these proteins in agricultural crops, especially in relation to natural processes like fruit ripening where ethylene plays a major role.

Banana is one of the most important fruit species in tropical and sub-tropical countries, ranking as the world’s second largest fruit crop and listing among the world’s ten most important food commodities (Sreedharan et al., 2012). Banana is a typical climacteric fruit, characterized by a burst in respiration and a typical increase in ethylene biosynthesis that initiates ripening-associated processes. This, from an economic perspective, limits fruit shelf-life with rapid deterioration of peel color and pulp firmness (Ba et al., 2016). For example, ripened bananas become unmarketable within 1–3 days at ambient temperature (Ahmed and Palta, 2016). Although numerous post-harvest practices such as low temperature storage, thermal processing, chemical, and biological treatments coupled with other preservation techniques are applied on fresh produces to maintain or extend the shelf-life, severe post-harvest losses still occur (Kuan et al., 2015). Therefore, a better understanding of the regulators involved in banana fruit ripening will help develop more effective post-harvest storage technologies. Since bananas are climacteric fruits, considerable effort has been directed to study genes involved in ethylene biosynthesis and signaling pathways including 1-aminocyclopropane-1-carboxylic acid (ACC) synthase (ACS), ACC oxidase (ACO), ethylene receptor, CTR1 ortholog, ethylene insensitive3 (EIN3)/EIN3-like (EIL), EIN3 binding F-box (EBF) and ERF genes (Liu et al., 1999; Mbéguié-A-Mbéguié et al., 2008; Kuang et al., 2013; Xiao et al., 2013; Jourda et al., 2014). Interestingly, opposing functions have been reported for banana ERF genes. For instance, among the fifteen ERF TFs that have been isolated from banana fruit, MaERF11 binds to *MaACS1* and *MaACO1* promoters to suppress their activities whereas MaERF9 activates *MaACO1* promoter activity (Xiao et al., 2013). Whilst DREB and ERF TFs belong to the AP2/ERF families, little is known about DREBs role in fruit ripening, especially those with EAR motif.

In this study, we identified a DREB TF with EAR motif, designated as MaDEAR1, which is a nucleus-localized transcriptional repressor. MaDEAR1 was ethylene- and ripening-inhibited, with reduced levels of histone H3 and H4 acetylation at its regulatory regions during fruit ripening. More importantly, MaDEAR1 binds to and represses promoters of several cell wall-modifying genes associated with fruit softening, including expansins (*MaEXP1/3*), polygalacturonase (*MaPG1*), xyloglucan endotransglycosylase/hydrolase (*MaXTH10*), pectate lyase (*MaPL3*), and pectin methylesterase (*MaPME3*). Our results suggest that MaDEAR1 may be acting as a negative regulator of cell wall-modifying genes, unraveling new information on EAR motif-containing DREB TFs in relation to fruit ripening.

## Materials and Methods

### Plant Materials and Treatments

Pre-climacteric banana (*Musa acuminata*, AAA group, cv. Cavendish) fruit at the 70–80% plump stage were obtained from a local commercial plantation near Guangzhou, southern China. Harvested fruit were separated into fingers, and fruit of uniform weight, shape, and maturity with no visual defects were selected, rinsed in tap water, and then air-dried before treatments were applied. The post-harvest treatments include a control (natural ripening), ethylene-induced ripening (100 μL L^-1^ ethylene, 18 h), and 1-methylcyclopropene (1-MCP)-delayed ripening (0.5 μL L^-1^ 1-MCP, 18 h), as described previously by Shan et al. (2012). All assessments were conducted using three biological replicates and fruit pulp of all samples were frozen in liquid nitrogen immediately after sampling, and stored at –80°C for further use.

Tobacco bright yellow 2 (BY-2) suspension cells were cultured and prepared as described by Kumagai-Sano et al. (2006). Tobacco (*Nicotiana benthamiana*) plants were grown under a 16-h light (25°C) and 8-h dark (22°C) photoperiod. Four- to six-week-old tobacco plants were used for transient assays.

### RNA Extraction, Gene Isolation, and Sequence Analysis

Frozen banana fruit pulp were ground in liquid nitrogen using a mortar and pestle. Total RNA was extracted using the hot borate method described by Wan and Wilkins (1994). Total RNA (∼1 μg) from each sample was treated with DNAse I digestion using an RNAse-free kit (Promega Madison, Fitchburg, WI, USA). The above DNA-free total RNA was then used as template for RT-PCR. The first-strand cDNA of the product was subjected to PCR amplification. According to gene annotation, bioinformatics and RNA sequencing analyses (D’Hont et al., 2012), one full-length *DREB* gene containing an EAR motif, with complete start and stop codons, termed *MaDEAR1* (GSMUA_Achr3T13190_001 in Banana Genome Hub, XP_009392127 in NCBI), was identified and selected from banana whole-genome sequence. This segment was cloned and sequenced. Alignments were carried out on ClustalX (version 1.83) and GeneDoc software, and a phylogenetic tree was constructed using the Neighbor–Joining method in the MEGA5 program.

### Quantitative Real-Time PCR (qRT-PCR) Analysis

All qRT-PCR analysis and synthesis of first-strand cDNA were performed as described previously (Chen et al., 2011; Shan et al., 2012). The sequences of all primers used for qRT-PCR analysis are listed in **Supplementary Table S1**. qRT-PCR was carried out on a Bio-Rad CFX96 Real-Time PCR System using the SYBR^®^Green PCR Supermix Kit (Bio-Rad Laboratories) following the manufacturer’s instructions. *MaRPS2* (ribosomal protein 2) was selected as a reference gene according to our previous study on the selection of reliable reference genes under different experimental conditions (Chen et al., 2011). All qRT-PCR reactions were normalized using Ct value corresponding to the reference gene. The relative expression levels of target gene were calculated with the formula 2^-ΔΔCT^. Three independent biological replicates were used in the analysis.

### Sub-cellular Localization of MaDEAR1 Protein

The coding sequences of *MaDEAR1* were amplified and cloned into the pEAQ-GFP vectors (kindly gifted by Dr. George P. Lomonossoff), then the fusion construct and positive control GFP vector were electroporated into the *Agrobacterium tumefaciens* strain GV3101 using Gene PulserXcell^TM^ Electroporation Systems (Bio-Rad, Hercules, CA, USA). The primers for construct development are listed in **Supplementary Table S1**. The *Agrobacterium* harboring MaDEAR1-GFP or the positive control was inoculated for 16 h at 28°C. Cells were pelleted, resuspended at OD600 = 0.1 in infiltration buffer [10 mM MgCl2, 10 mM MES (pH 5.6), 100 μM acetosyringone], incubated for 4 h at room temperature, then was infiltrated into the abaxial side of 4- to 6-week-old tobacco leaves using a 1-mL needleless syringe as described previously by Sainsbury et al. (2009). Two days after infiltration, GFP fluorescence signals were observed by a fluorescence microscope (Zeiss Axioskop 2 Plus) with a beam splitter for excitation at 500 nm. All assays were repeated at least three times.

### Promoter Isolation and Analysis

Genomic DNA was extracted from banana leaves using the DNeasy Plant Mini Kit (Qiagen). The promoters of *MaDEAR1*, and genes including *MaEXP1/3*, *MaPG1*, *MaXTH10*, *MaPL3*, and *MaPME3* involved in cell wall loosening of banana fruit associated with softening (Pua et al., 2001; Asif and Nath, 2005; Asha et al., 2007; Mbéguié-A-Mbéguié et al., 2009; Asif et al., 2014), were isolated using a Genome Walker Kit (Clontech) with nested PCR according to the manufacturer’s instructions (specific primers are listed in **Supplementary Table S1**). After sequencing, conserved *cis*-element motifs of promoters were predicted using Plant-CARE^1^ databases.

### Promoter Activity Assay

The MaDEAR1 promoter region was amplified by PCR using the specific primers listed in **Supplementary Table S1**. The PCR product was inserted into the pGreenII 0800-LUC double reporter vector (Hellens et al., 2005) at the *Kpn*I and *Nco*I sites to fuse it with the Firefly luciferase (LUC) reporter gene (MaDEAR1 pro-LUC). A Renilla luciferase (REN) under the control of the 35S promoter at the same vector was used as an internal control. The construct CaMV35S-REN/*MaDEAR1* pro-LUC (∼20 μg) was transformed into tobacco BY-2 protoplasts (∼2 × 10^4^) by polyethylene glycol (PEG) methods as described previously (Abel and Theologis, 1994).

The promoter activity was assayed according to Ba et al. (2014a). The transformed protoplasts were subjected to 0 mM (control) or 0.8 mM of ethrel (ethylene releaser) treatment and then incubated at 23°C for 14 h, and LUC and REN activities were assayed using the dual luciferase assay kits (Promega), and the promoter activity is indicated by the ratio of LUC to REN. The analysis was carried out using the Luminoskan Ascent Microplate Luminometer (Thermo) according to the manufacturer’s instructions, with a 5-s delay and 15-s integrated measurements. At least six assay measurements were included for each.

### Chromatin Immunoprecipitation (ChIP) and Quantitative PCR Analysis

Chromatin immunoprecipitation (ChIP) was performed as described earlier (Gendrel et al., 2005; Benhamed et al., 2006). Unripe and ripe banana fruit pulp were collected and crosslinked in 1% formaldehyde for 15 min in a vacuum and then neutralized by 0.125 M glycine. After washing with sterilized water, 5 g of banana fruit pulp were ground in liquid nitrogen, and suspended in a buffer containing 0.25 M sucrose, 10 mM Tris-HCl (pH 8.0), 10 mM MgCl_2_, 1% Triton X-100, 5 mM β-mercaptoethanol, 0.1 mM PMSF, and protease inhibitors (one minitablet per milliliter; Roche). The suspensions were transferred to microfuge tubes and centrifuged at 12,000 *g* for 10 min. The pellets were suspended in 1.7 M sucrose, 10 mM Tris-HCl (pH 8.0), 2 mM MgCl_2_, 0.15% Triton X-100, 5 mM β-mercaptoethanol, 0.1 mM PMSF, and protease inhibitors and centrifuged through a layer of the same buffer in microfuge tubes. The chromatin extracts were lysed in a buffer containing 50 mM Tris-HCl, pH 8, 10 mM EDTA, 1% SDS, and protease inhibitors, and were sheared to an average length of 500 bp by sonication at 4°C using the Sonics VCX800 apparatus followed by centrifugation. The supernatants were diluted 10-fold with 1% Triton X-100, 1.2 mM EDTA, 16.7 mM Tris-HCl (pH 8.0), and 167 mM NaCl. A 1 mL aliquot of the dilution was immunoprecipitated with specific antibodies of anti-acetyl-histone H3 (anti-H3ac) and anti-acetyl-histone H4 (anti-H4ac) (Millipore), while immunoglobulin G (IgG) was used as a negative control. ChIP assays were repeated with three biological replicates. The DNA cross-linked to immunoprecipitated proteins and input DNA were detected by qRT-PCR. *MaACT2* (GSMUA_Achr9G03170_001) was used as internal control since its histone acetylation level is stable during ripening (Han et al., 2016). The percentage of IP/Input was calculated by determining 2^-ΔCt^ (=2^-[Ct(IP)-Ct(Input)]^). The primers used for ChIP-qPCR analysis are listed in **Supplementary Table S1**.

### Protein Expression and Electrophoretic Mobility Shift Assay (EMSA)

*MaDEAR1* was cloned into pGEX-4T-1 (Amersham Biosciences) to fuse in frame with GST and expressed in BM Rosetta (DE3) by induction with 1 mM of isopropyl-β-D-thiogalactopyramoside (IPTG) for 6 h at 30°C. The recombinant protein was purified with Glutathione Sepharose 4B (GE Healthcare). The EMSA was performed using the EMSA kit (Thermo) according to the manufacturer’s instructions. The probes containing the DRE/CRT (core sequence for A/GCCGAC) element (2 × 10^-6^ μmol) derived from *MaEXP1/3*, *MaPG1*, *MaXTH10*, *MaPL3*, and *MaPME3* promoters were labeled with biotin using DNA 3′ End Biotinylation Kit (Thermo). The same unlabeled DNA fragment with 2 × 10^-5^ μmol, 2 × 10^-4^ μmol, or 2 × 10^-3^ μmol, respectively, was used as a competitor. After cross-linking, the membrane was detected by the chemiluminescence method according to the manufacturer’s protocol on a ChemiDoc^TM^ MP Imaging System (Bio-Rad). The primers used in protein expression and EMSA assays are listed in **Supplementary Table S1**.

### Transient Assay in Tobacco Leaves

A dual-luciferase reporter system was used in the transient assay, and all primers used for the following constructs are listed in **Supplementary Table S1**. For transcriptional activity analysis of MaDEAR1, the coding sequence of *MaDEAR1* was inserted into the constructed pBD vector driven by the 35S promoter as the effector, and the double reporter vector includes a native GAL4-LUC, and an internal control REN driven by 35S promoter, which was modified based on pGreenII 0800-LUC reporter vector (Hellens et al., 2005). GAL4-LUC contains five copies of GAL4 binding element and 35S promoter, and these sequences are located upstream of the LUC. For the assay of MaDEAR1 repressing the *MaEXP1/3*, *MaPG1*, *MaXTH10*, *MaPL3*, and *MaPME3* promoters, *MaDEAR1* were inserted into the pEAQ vector as effector, while the promoters were cloned into pGreenII 0800-LUC double-reporter vector as reporter.

The constructed effector and reporter plasmids were co-transformed into tobacco leaves by *Agrobacterium tumefaciens* strain GV3101. LUC and REN luciferase activities were measured as described above. The transcriptional activity of MaDEAR1 and the binding activity of MaDEAR1 to the promoter are indicated by the ratio of LUC to REN. At least six biological replicates were assayed for each combination.

### Statistical Analysis

Experiments were conducted using a completely randomized design. Each sample time point for each treatment comprised three independent biological replicates. Data were plotted as means ± standard errors (SE) in figures. Least significant difference (LSD) at the 5% level was estimated using DPS software (version 3.01; Zhejiang University, Hangzhou, China).

## Results

### MaDEAR1 Is an A-5 Sub-group Member of the DREB Family

**FIGURE 1:**
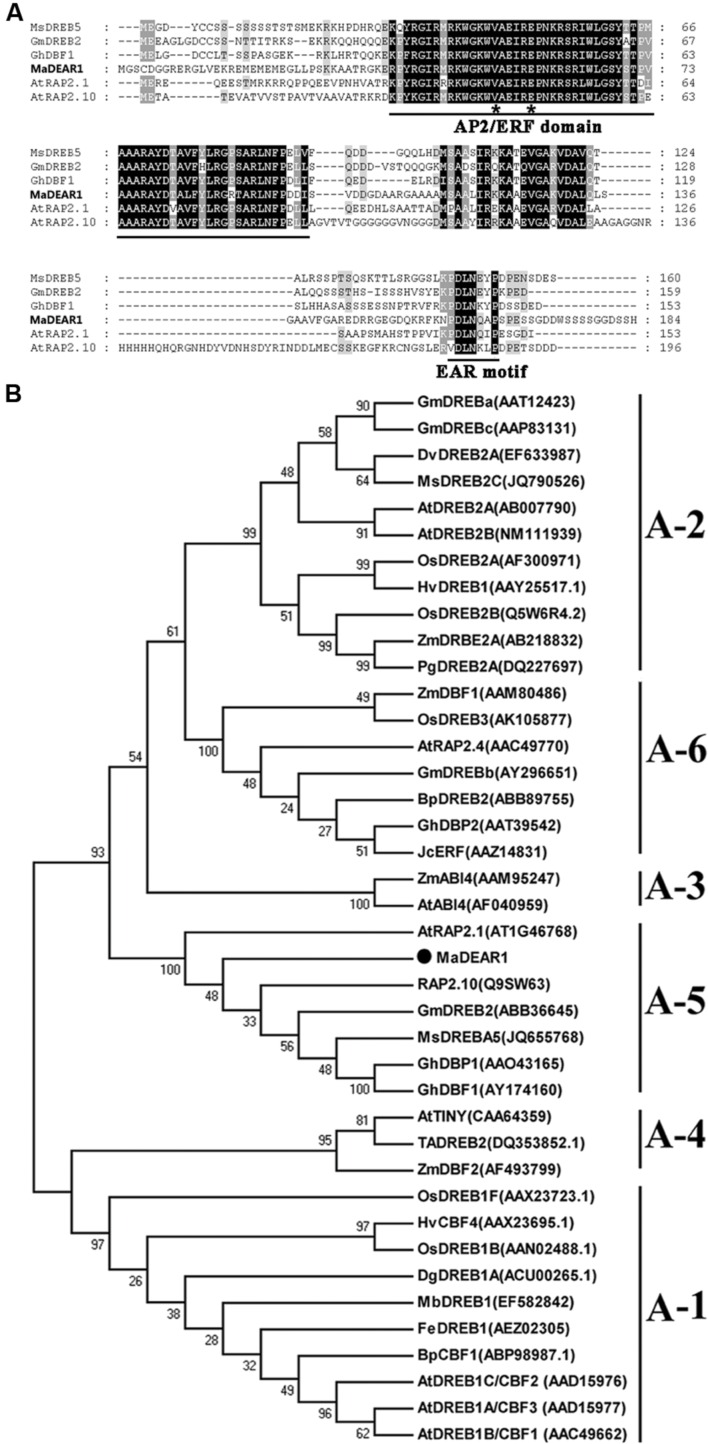
**Sequence analysis of MaDEAR1.** **(A)** Alignment of the deduced amino acid sequences of the banana MaDEAR1 and its respective homologs MsDREBA5 (JQ655768), GmDREB2 (DQ208968), AtRAP2.1 (AT1G46768), and AtRAP2.10 (AT4G36900). Identical and similar amino acids were presented by black and gray shading, respectively. The AP2/ERF domain and the EAR-motif (DLNXXP) were underlined. The 14th and 19th amino acids were indicated by asterisks. **(B)** Phylogenetic analysis of plant DREB family proteins. The multiple alignment was made using ClustalW, and the phylogenetic tree was constructed with MEGA 5.0 using a bootstrap test of phylogeny with minimum evolution test and default parameters. Numbers indicate bootstrap values.

Based on gene annotation, bioinformatics and RNA sequencing analyses (D’Hont et al., 2012), one full-length *DREB* gene containing an EAR motif, designated as *MaDEAR1* (*M. acuminata* DREB and EAR motif protein 1) (GSMUA_Achr3T13190_001 in Banana Genome Hub, XP_009392127 in NCBI), which was found down-regulated during fruit ripening, did attract our attention. MaDEAR1 encodes a protein of 184 amino acids, with calculated molecular weight of 20.24 kDa and *p*I value of 10.10. Analysis of deduced amino acid sequence of MaDEAR1 revealed a typical AP2/ERF domain of 58 amino acids with the conserved valine (V) and glutamic acid (E) at the 14th and 19th positions, respectively (**Figure 1A**), which are considered to be essential sites for the binding of DREBs to the DRE *cis*-elements (Sakuma et al., 2002). In addition, a repressor domain, the ERF-associated amphiphilic repression (EAR) motif was observed at the C-terminus of the protein (**Figure 1A**), suggesting that the MaDEAR1 might function as a transcriptional repressor. Phylogenetic analyses showed that DREB proteins can be classified into six sub-groups (A1–A6), in which MaDEAR1 together with AtRAP2.1, GmDREB2, GhDBP1, and MsDREB5 belong to A-5 sub-group (**Figure 1B**). Collectively, these data suggest that MaDEAR1 is an A-5 sub-group member of the DREB family.

### MaDEAR1 Is a Nucleus-Localized Transcriptional Repressor

**FIGURE 2:**
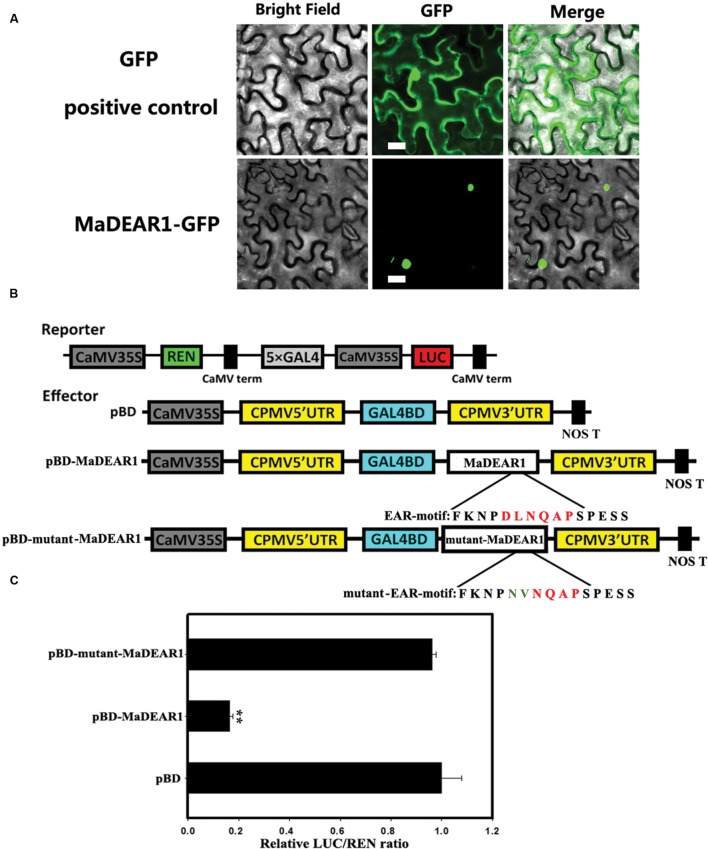
**Sub-cellular localization and transcriptional repression activity of MaDEAR1.** **(A)** MaDEAR1 is localized in nucleus. *Agrobacterium tumefaciens* carrying MaDEAR1-GFP or GFP positive control were infiltrated into tobacco leaves. After 48 h, the fluorescence of MaDEAR1 protein was localized exclusively in the nucleus, while the fluorescence of the GFP positive control was distributed in both nucleus and cytoplasm. Bar, 20 μM. **(B)** Reporter and effector constructs. The dual luciferase reporter construct contained the LUC reporter gene fused with 5 × GAL4 and CaMV35S. The effector plasmid contained the *MaDEAR1* gene or with mutant EAR motif fused to GAL4BD driven by the CaMV35S. The two conserved amino acids (DL) of the EAR-motif without or with site-mutation (NV) are also shown. pBD was used as a negative control. **(C)** Transcriptional repression ability of MaDEAR1 *in vivo*. Compared with the pBD control, pBD-MaDREB5 significantly repressed the expression of the LUC reporter. The ratio of LUC to REN of the pBD vector was used as a calibrator (set as 1). Each value represents the means of six biological replicates, and vertical bars represent the SE Asterisks indicate a statistically significant difference compared with pBD by Student’s *t*-test. ^∗∗^*P* < 0.01.

To examine the sub-cellular localization of MaDEAR1 *in vivo*, we fused the *MaDEAR1* coding region in-frame with the N-terminal side of green fluorescent protein (GFP) under the control of CaMV 35S promoter. The MaDEAR1-GFP and GFP control plasmids were transiently expressed in tobacco leaves by *Agrobacterium* infiltration. While control GFP accumulated in both nucleus and cytoplasm, the MaDEAR1-GFP fusion protein was clearly localized in the nucleus (**Figure 2A**). This suggests that MaDEAR1, like other reported DREB proteins, is a nuclear protein which is a typical feature of TFs.

To investigate whether MaDEAR1 possesses transcriptional repression activity *in vivo*, a dual luciferase assay was performed. The dual-luciferase reporter harbors five copies of the GAL4 DNA-binding element and CaMV 35S fused to the firefly luciferase (LUC) reporter, whereas a renilla luciferase (REN) reporter under the control of the 35S promoter was used as an internal control. Full-length *MaDEAR1* was fused with GAL4 DNA-binding domain (GAL4-BD) as the effector, and the empty GAL4-BD (pBD-empty) was used as a negative control (**Figure 2B**). As shown in **Figure 2C**, compared with the pBD-empty control, pBD-MaDEAR1 significantly repressed the expression of the LUC reporter, with approximately fivefold less LUC/REN value than the control. To further confirm whether the conserved EAR motif (DLNQAP) was important for the MaDEAR1-mediated repression, site-specific mutations were made to convert two conserved amino acids (DL) to NV (**Figure 2B**). As expected, the transcriptional repression ability of MaDEAR1 was abolished when the EAR-motif was mutated (**Figure 2C**). These results confirm that MaDEAR1 may act as a transcriptional repressor, and the EAR motif is important for its repression activity.

### *MaDEAR1* Is Inhibited by Ethylene and Ripening

**FIGURE 3:**
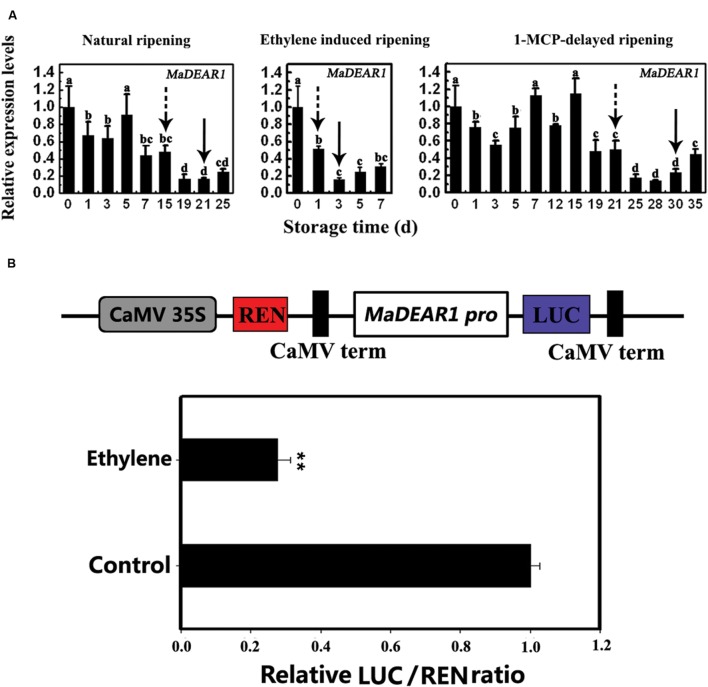
***MaDEAR1* is inhibited by ethylene and ripening.** **(A)** Expression of *MaDEAR1* in pulp during three ripening conditions, which include natural (control), ethylene-induced, and 1-MCP-delayed ripening. The expression levels of *MaDEAR1* are expressed as a ratio relative to the harvest time (0 days of control), which was set at 1. Each value represents the mean ± SE of three biological replicates. Different letters above bars indicate significant difference at the 5% level by Student’s *t*-test. The broken arrow and full arrow indicate the time point at which ethylene production began to increase and its peak for each treatment, respectively. The physiological data such as changes in fruit firmness and ethylene production during banana fruit ripening and softening have been presented in Shan et al. (2012). **(B)** *MaDEAR1* promoter activity in response to ethylene. The dual luciferase reporter vector containing *MaDEAR1* promoter (CaMV35S–REN/*MaDEAR1* pro-LUC) was transiently transformed into tobacco BY-2 protoplasts using a modified PEG method, and the transformed protoplasts were subjected to 0 (control) or 0.8 mM ethrel (ethylene releaser) treatment. After incubation for 14 h, LUC and REN luciferase activities were assayed, and the promoter activity is indicated by the ratio of LUC to REN. Each value represents the means of six biological replicates, and vertical bars represent the SE ^∗∗^*P* < 0.01 by Student’s *t*-test.

Our previous study showed that fruits in natural ripening group start ethylene production after storage at 22°C and 90% relative humidity for 15 days, which peaks around day-21, and declines thereafter. Ethylene-treated fruit ripened rapidly, with an ethylene peak appearing at day 3 following treatment. In contrast, 1-MCP treatment delayed ripening, with ethylene peaking at day 30 (Shan et al., 2012). To investigate the expression of *MaDEAR1* during banana fruit ripening, we performed quantitative RT-PCR analysis using banana fruit in three different ripening behaviors caused by natural, ethylene-induced, and 1-MCP-delayed ripening treatments. As shown in **Figure 3A**, *MaDEAR1* expression was repressed by ethylene, and its transcript level in natural, ethylene-induced or 1-MCP-delayed ripening was decreased following ethylene production appearance, revealing that *MaDEAR1* expression was suppressed by ethylene and ripening.

To better understand the mechanism(s) of *MaDEAR1* expression modulation, a 976 bp upstream sequence from the start codon of *MaDEAR1* was isolated from the genome of *M. acuminata* using a genome-walking PCR method. Analysis of the promoter using the PLACE and Plant-CARE databases revealed a site for the ethylene-responsive element (ERE), ATTTCAAA, with one nucleotide change found in the promoter at –507 to –515 bp from the initiation codon (**Supplementary Data Sheet S1**), indicating that *MaDEAR1* promoter might respond to ethylene. We then fused the *MaDEAR1* promoter in front of LUC in the dual luciferase reporter vector, while the REN driven by the CaMV 35S promoter at the same vector was used as an internal control (**Figure 3B**). The resultant vector was transiently expressed in tobacco BY2 protoplasts with or without ethrel treatment, and the luciferases were assayed thereafter. As shown in **Figure 3B**, after the treatment with ethrel, the promoter activity of *MaDEAR1* was dramatically decreased, as evidenced by the much lower relative LUC/REN ratio compared that of the control, indicating that *MaDEAR1* promoter activity was suppressed by ethylene.

### Decrease Expression of *MaDEAR1* Is Correlated with Histone Acetylation Changes during Fruit Ripening

**FIGURE 4:**
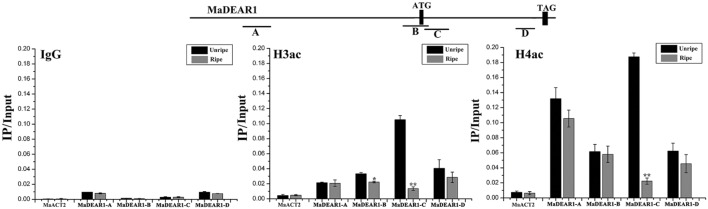
**ChIP-qPCR analysis of histone H3 (H3ac) or H4 acetylation (H4ac) levels at the regions of *MaDEAR1* in unripe and ripe banana fruit.** The four regions of *MaDEAR1* (A–D) examined by ChIP-qPCR are shown in the top panel. The amounts of DNA after ChIP were quantified and normalized to *MaACT2*. IgG and *MaACT2* were used as negative controls. Data are shown as the ratio of IP to input (IP/Input). Each experiment was repeated with three biological replicates. Error bars represent SE ^∗^*P* < 0.05 and ^∗∗^*P* < 0.01 by Student’s *t*-test, compared with unripe fruit.

Histone acetylation is a type of chromatin modification facilitating the gene expression, which is closely associated with gene activation. To assess whether the expression of *MaDEAR1* during fruit ripening is associated with histone acetylation, we examine the histone acetylation levels of *MaDEAR1* in fruit of unripe and ripe stages by ChIP-qPCR assays using antibodies such as anti-acetyl-histone H3 (H3ac) and anti-acetyl-histone H4 (H4ac). As shown in **Figure 4**, as a negative control, the enrichments of IgG in the promoter and coding region of *MaDEAR1* were low. In addition, no significant difference of histone H3 or H4 acetylation levels of *MaACT2* between unripe and ripe banana fruit was observed. On the contrary, the histone H3 acetylation (H3ac) levels of *MaDEAR1* in region B and C as well as histone H4 acetylation (H4ac) levels of *MaDEAR1* in region C were decreased in ripening bananas (**Figure 4**), which is consistent with its decreased level of expression during ripening (**Figure 3A**). These findings, together with the observations of its gene expression and promoter activity, suggest that *MaDEAR1* is suppressed by ethylene and ripening, and its decreased expression might be associated with reduced levels of histone H3 and H4 acetylation during ripening.

### Genes Involving in Cell Wall Loosening of Banana Fruit, *MaEXP1/3*, *MaPG1*, *MaXTH10*, *MaPL3*, and *MaPME3*, Are Direct Targets of MaDEAR1

To understand the possible roles of MaDEAR1 in banana fruit ripening, we identified the potential targets of MaDEAR1 in relation to fruit ripening. Previous studies indicated that DREB proteins bind to the *cis*-acting dehydration-responsive element/C-repeat (DRE/CRT) in the promoter of their target genes, including *RD29A*, *RD17*, *COR15A*, *ERD10*, *KIN1*, and *COR6.6* (Lucas et al., 2011). Softening is an important indicator of fruit ripening, which is related to cell wall modifications, including enzymatic and non-enzymatic degradation of cell wall components (Li et al., 2010). Previously, 23 cell wall-modifying genes, including five *EXP*, nine *XET/XTH*, four *PG*, two *PE/PL* and three *PME* have been reported to be related to banana fruit softening (Pua et al., 2001; Asif and Nath, 2005; Asha et al., 2007; Mbéguié-A-Mbéguié et al., 2009; Asif et al., 2014), and we have examined the presence of the DRE/CRT core consensus sequence (A/GCCGAC) in their promoters. It was found that *MaEXP1*, *MaXTH10*, *MaPME3*, *MaPG1*, and *MaPL3* have one or two DRE/CRT elements in their promoters, while *MaEXP3* promoter contained four DRE/CRT elements (**Supplementary Data Sheet S1**), suggesting that these genes might be the targets of MaDEAR1.

**FIGURE 5:**
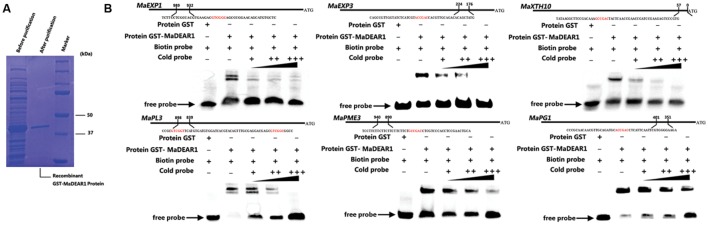
**MaDEAR1 binds to promoters of cell wall-modifying genes.** **(A)** SDS-PAGE gel stained with Coomassie blue demonstrating affinity purification of the recombinant MaDEAR1 protein used for the electrophoretic mobility shift assay (EMSA). **(B)** EMSA showing MaDEAR1 binding to the promoter of *MaEXP1/3*, *MaPG1*, *MaXTH10*, *MaPL3*, and *MaPME3* containing DRE/CRT *cis*-acting element. Biotin-labeled DNA probe from the promoter was incubated with GST-MaDEAR1 protein, and the DNA-protein complexes were separated on 6% native polyacrylamide gels. Triangles indicate increasing amounts of unlabeled probes (2 × 10^-5^ μmol, 2 × 10^-4^ μmol, or 2 × 10^-3^ μmol) for competition.

To test whether MaDEAR1 could directly bind to these promoters, electrophoretic mobility shift assay (EMSA) was performed. DNA fragments containing the DRE/CRT element in the region of these promoters were used as probe. Recombinant glutathione S-transferase (GST)-MaDEAR1 fusion proteins were expressed in *E. coli* and purified (**Figure 5A**). As shown in **Figure 4B**, MaDEAR1 can directly bind to the DNA probes containing the DRE-motif in *MaEXP1/3*, *MaPG1*, *MaXTH10*, *MaPL3*, and *MaPME3* promoters, and the bindings were abolished by the addition of increasing amounts of unlabeled competitors with the same sequence. In addition, no parallel band shift was detected with only the GST tag (**Figure 5B**). These results show that MaDEAR1 specifically binds to the DRE motif in the promoters of *MaEXP1/3*, *MaPG1*, *MaXTH10*, *MaPL3*, and *MaPME3*, demonstrating that these genes are likely direct targets of MaDEAR1.

### MaDEAR1 Represses Promoter Activities of *MaEXP1/3*, *MaPG1*, *MaXTH10*, *MaPL3*, and *MaPME3*

**FIGURE 6:**
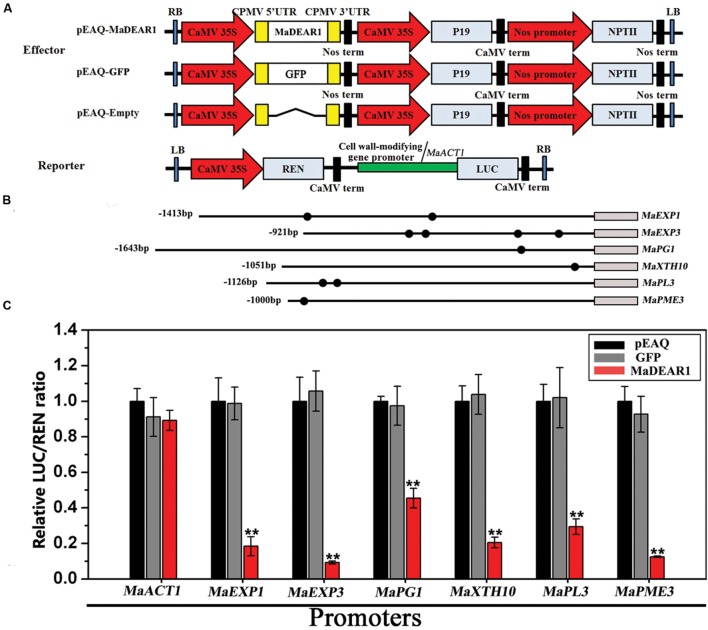
**Transient dual-luciferase reporter assays showing MaDEAR1’s ability to repress the promoter activity of *MaEXP1/3*, *MaPG1*, *MaXTH10*, *MaPL3*, or *MaPME3*.** **(A)** Constructs used in the transient assays. The reporter contained the *MaEXP1/3*, *MaPG1*, *MaXTH10*, *MaPL3*, or *MaPME3* promoters fused to LUC luciferase and REN luciferase driven by CaMV 35S as internal control. The effector contained the MaDEAR1 driven by the CaMV35S. The effector vector also contained the P19 suppressor of gene silencing, and the NPTII kanamycin resistance gene. **(B)** Schematics of the promoter of *MaEXP1/3*, *MaPG1*, *MaXTH10*, *MaPL3*, or *MaPME3*. Promoter length and DRE/CRT *cis*-acting elements are indicated with lines and black circles, respectively. **(C)** MaDEAR1 represses the promoter activity of *MaEXP1/3*, *MaPG1*, *MaXTH10*, *MaPL3*, or *MaPME3*. *Agrobacterium tumefaciens* strain GV3101 carrying the LUC reporter plasmid and different combinations of effector plasmids was infiltrated into *N. benthamiana* leaves, and the luciferase activity at the site of infiltration was measured 2 days after infiltration. The repression ability of MaDEAR1 to the promoter was shown by the ratio of LUC to REN. The ratio of LUC to REN of the empty vector (pEAQ) plus promoter was used as a calibrator (set as 1). The reporter containing the *MaACT1* promoter, and effector carrying the *GFP* gene were used as negative controls. Each value represents the means of six biological replicates, and vertical bars represent the SE ^∗∗^*P* < 0.01 by Student’s *t*-test, compared with pEAQ.

To further understand the regulation of *MaEXP1/3*, *MaPG1*, *MaXTH10*, *MaPL3*, and *MaPME3* by MaDEAR1, we performed transient expression assays using the dual luciferase reporter system. The dual luciferase reporter plasmid harbor *MaEXP1/3-*, *MaPG1-*, *MaXTH10-*, *MaPL3-*, or *MaPME3-*promoter fused to LUC, and the REN driven by the CaMV 35S promoter, while an effector plasmid carried MaDEAR1 expressed under the control of the CaMV 35S promoter (**Figure 6A**). As shown in **Figure 6B**, compared with the negative controls, tobacco leaves expressing MaDEAR1 showed significantly lower luciferase activity with the constructs harboring the LUC reporter gene driven by the *MaEXP1/3-*, *MaPG1-*, *MaXTH10-*, *MaPL3-*, or *MaPME3-*promoter, which is consistent with the finding that MaDEAR1 is a transcriptional repressor (**Figure 2B**).

## Discussion

Dehydration-responsive element-binding proteins are a class of AP2/ERF family of TFs, which are involved in plant response to drought, high salinity, low-temperature, and other environmental stresses (Agarwal et al., 2006; Lata and Prasad, 2011). The DREB proteins are divided into six small groups (A-1 to A-6) based on the sequence signature of DNA-binding domain and the existence of other motifs, of which A-5 group members share a conserved EAR motif at their C-terminus and act as transcriptional repressors (Ohta et al., 2001; Sakuma et al., 2002). In this study, we identified a banana *DREB* gene, *MaDEAR1*, whose predicted protein possesses an APETALA2 (AP2) domain that binds to DREs and an EAR motif that is responsible for transcriptional repression (**Figures 1A** and **2**), and thus belongs to A-5 group (**Figure 1B**). Sub-cellular localization and transcriptional activation assays indicated that MaDEAR1 is nuclear-localized and possesses transcriptional repression activity (**Figure 2**), similar to GhDBP1 in cotton (Huang and Liu, 2006) and MsDREBA5 in *Malus sieversii* Roem (Zhao et al., 2012).

To date, most reports about DREBs focus on A-1 and A-2 sub-groups, while investigation into others, such as the A-5 sub-group, is limited. Generally, the A-5 sub-group DREB proteins are transcriptional repressors of gene expression during stress. For example, transgenic *Arabidopsis* over-expressing *DEAR1* showed a cell death phenotype, resulting in reduced freezing tolerance (Tsutsui et al., 2009). Similarly, mutations in *RAP2.1*, another A-5 DREB, led to increased expression of *DREB1/CBF* and *DREB2* target genes with enhanced tolerance to drought and freezing (Dong and Liu, 2010). More recently, over-expression of *TaRAP2.1L* (a homolog of *RAP2.1* in wheat) under constitutive and stress-inducible promoters in transgenic wheat and barley caused dwarfism and decreased frost tolerance, supporting the notion that most DREB members in A-5 sub-group are negative regulators of stress tolerance (Amalraj et al., 2016). However, in addition to their role in stress responses, whether A-5 DREBs are involved in other biological processes such as fruit ripening is unknown. Here we showed that the *MaDEAR1* expression was down-regulated by ethylene and ripening. The ripening associated down-regulation is, at least in part, likely to be mediated by the repression of its promoter activity by ethylene produced during ripening (**Figure 3**). It is also important to note that the decline in *MaDEAR1* expression showed a concomitant increase in ethylene production during fruit ripening. These findings suggest that MaDEAR1 is a transcriptional repressor associated with fruit ripening, which is consistent with its transcriptional repression activity reported here (**Figure 2B**). Interestingly, we also found reduced levels of histone H3ac and H4ac in *MaDEAR1* promoter in the ripening stage of banana fruit (**Figure 4**), which closely corresponds with its decreased expression during fruit ripening (**Figure 3**). Several reports have implicated histone acetylation as an important mechanism for controlling *DREB* gene expression. For example, treatment of maize with the HDAC inhibitor trichostatin A (TSA) under cold stress conditions selectively inhibited the induction of the cold-responsive gene *ZmDREB1* through histone modification in the promoter region (Hu et al., 2011). Reports on enhanced transcript level of rice *OsDREB1b* and maize *ZmDREB2A*, as well as histone acetylation in their promoters further attest the regulation of *DREB* gene expression through histone modification (Roy et al., 2014; Zhao et al., 2014).

Fruit softening is one of the most important features that characterize the ripening process of fleshy climacteric fruits like bananas, and cell wall modification that occurs during the ripening process plays a critical role in the softening process (Li et al., 2010). The process of fruit ripening involves enzymatic and non-enzymatic factors. Expansins are non-enzymatic cell wall proteins that primarily induce cell wall extension (Cosgrove, 2000). Secondary wall-loosening factors such as XTH and PG, which enzymatically modify the structures of the cell wall, render it more responsive to wall-loosening events mediated by expansins (Cosgrove, 2000; Péret et al., 2009). XTH proteins can display two distinct enzymatic activities, including transglycosylase enzymatic (XET) activity leading to xyloglycan chain synthesis, and xyloglucan hydrolase activity (XEH) resulting in their degradation (Saladie et al., 2006). According to the previous reports, 23 cell wall-modifying genes, including five *EXP*, nine *XET/XTH*, four *PG*, two *PE/PL* and three *PME* have been isolated from banana fruit (Pua et al., 2001; Asif and Nath, 2005; Asha et al., 2007; Mbéguié-A-Mbéguié et al., 2009; Asif et al., 2014). It was found that transcripts of these genes were differentially expressed during post-harvest ripening (Mbéguié-A-Mbéguié et al., 2009; Asif et al., 2014), supporting their involvement in fruit ripening and softening. It is worth noting that transcriptional regulation of cell wall-modifying genes might be a conserved mechanism by which TFs regulate fruit ripening, as the cases of RIN in tomato (Fujisawa et al., 2013) and AdEILs and AdERFs in kiwifruit (Yin et al., 2010). Similar results were also found in bananas. For instance, MaMADS5 binds to the CArG-box sequence in the promoters of several ripening genes including *MaEXPs* (Roy Choudhury et al., 2012). Our previous studies also indicated that MaLBDs and MaBSD1 TFs are involved in fruit ripening, via transcriptional regulation of *MaEXP1/2* (Ba et al., 2014a,b). Also we found that, a transcriptional repressor MaDof23 physically interacts with a transcriptional activator MaERF9, and they act antagonistically to regulate 10 ripening-related genes including *MaEXP1/2/3/5*, *MaXET7*, *MaPG1*, *MaPME3*, *MaPL2*, *MaCAT*, and *MaPDC* that are associated with cell wall degradation and aroma formation during banana ripening (Feng et al., 2016). In this work, we show that MaDEAR1 binds and represses the activity of six cell wall-modifying genes, such as *MaEXP1/3*, *MaPG1*, *MaXTH10*, *MaPL3*, and *MaPME3* (**Figures 5** and **6**), suggesting its regulatory role in cell wall degradation during banana fruit ripening. Nuclear localization and promoter activity of MaDEAR1 in banana protoplasts will further substantiate these results. In addition to these fruit softening-associated genes, those involved in ethylene production and aroma formation such as *MaACS1*, *MaACO1* and *MaPDC* are also associated with banana ripening (Yang et al., 2011; Xiao et al., 2013), but whether they are direct targets of MaDEAR1 remains unknown. As fruit ripening is controlled by transcriptional regulatory networks involving several TFs, it would be interesting to investigate whether MaDEAR1 interacts with other reported ripening-related TFs of banana fruit, including MaERFs (Xiao et al., 2013), MaMADSs (Roy Choudhury et al., 2012), MaNACs (Shan et al., 2012), MaLBDs and MaBSDs (Ba et al., 2014a,b), and the effects of such interactions on fruit ripening.

## Conclusion

The data reported here represent an EAR-motif-containing DREB TF, MaDEAR1, which was found to be a nuclear-localized transcriptional repressor. Expression and promoter activity of *MaDEAR1* were repressed by ethylene and ripening, and its expression is likely to be regulated by, at least in part, by histone modification. MaDEAR1 binds to and represses several cell wall-modifying genes, including *MaEXP1/3*, *MaPG1*, *MaXTH10*, *MaPL3*, and *MaPME3*. Taken together, our results report new insights into the mechanisms underpinning ethylene-mediated banana fruit ripening, of which MaDEAR1 may be playing a role via transcriptional repression of genes involved in cell wall modification and softening of fruit.

## Author Contributions

JC, JK, WL, PL, and XD designed the research. ZF, WS, YH, YX, and YY performed the experiments. ZF, JK, WL, PL, and JC wrote the manuscript.

## Conflict of Interest Statement

The authors declare that the research was conducted in the absence of any commercial or financial relationships that could be construed as a potential conflict of interest.

## Footnotes

1 http://bioinformatics.psb.ugent.be/webtools/plantcare/html/
