# Supplementary material for: The Banana Transcriptional Repressor MaDEAR1 Negatively Regulates Cell Wall-Modifying Genes Involved in Fruit Ripening
Source: Front Plant Sci. 2016 Jul 11;7:1021. doi: 10.3389/fpls.2016.01021 (PMC4939300; doi:10.3389/fpls.2016.01021)
Supplement: Supplementary file 1 [file Table_1.PDF]

Supplementary Table 1 Summary of primers used in this study.

Assay	Primer sequences (5' to 3')		Restriction Site
**RT-qPCR**	*MaDEAR1-For*	GAGGGAGAGAGGTCTGGTAG	
	MaDEAR1-Rev	AAGTTGAGGCGAGCGGTGC	
**Subcellular localization**	*MaDEAR1*-GFPFor	CAAATTCGCGaccggt ATGGGATCATGCGATGGAGG	AgeI
	MaDEAR1-GFPRev	TGCTAGTCATaccggtCTAGTGCGAGCTGTCGCCGCC	AgeI
**EAR motif mutation**	*MaDEAR1-For*	ATGGAGATGGAGGGGCTGTT	
	MaDEAR1-Rev	CTAGTGCGAGCTGTCGCCG	
	mutant-MaDEAR1-For	GAATCCTAATGTCAACCAGG	
	mutant-MaDEAR1-Rev	GGTTGACATTAGGATTCTTGA	
**ChIP-qPCR analysis**	*MaDEAR1-AFor*	CACTCGACCCATTCCGCA	
	MaDEAR1-ARev	TACGAGACGGTTGTTGAGATGC	
	MaDEAR1-BFor	GCCCTCCAAATGTGCAGCCAA	
	MaDEAR1-BRev	CCAGACCTCTCTCCCTCCCTC	
	MaDEAR1-CFor	GGAGGGGCTGTTGCCATCG	
	MaDEAR1-CRev	GGAGTAGGAGCCGAGCCAT	
	MaDEAR1-DFor	CAACTTCCCCGACGACATCT	
	MaDEAR1-DRev	AGATCAGGATTCTTGAACCGTTT	
**Fusing GST**	pGEX-4T-1-*MaDEAR1-For*	GGTTCCGCGTggatcc ATGGGATCATGCGATGGAGG	BamHI
	pGEX-4T-1-*MaDEAR1-Rev*	AGTCACGATgcggccgcCTAGTGCGAGCTGTCGCCGCC	NotI
**Dual LUC assay**	*pBD- MaDEAR1-For*	TCGCCGACCGGTaggcctATGGGATCATGCGATGGAGG	Stu I
	pBD- MaDEAR1-Rev	AACCAGAGTTAAaggcctCTAGTGCGAGCTGTCGCCGCC	Stu I
	pEAQ-MaDEAR1-1For	CAAATTCGCGaccggtATGGGATCATGCGATGGAGG	AgeI
	pEAQ-MaDEAR1-1Rev	AGTTAAAGGCctcgagCTAGTGCGAGCTGTCGCCGCC	XhoI
	0800- MaDEAR1proFor	CTATAGGGCGAATTGggtaccTCACTCGACCCATTCCGCAC	KpnI
	0800-MaDEAR1proRev	TATGTTTTTGGCGTCTTccatCTCCATCTCTCTCTTCTCTACC	NcoI
	0800-MaEXP1proFor	CTATAGGGCGAATTGggtaccCGCACAGCGTCCTCAAGCGG	KpnI
	0800-MaEXP1proRev	TATGTTTTTGGCGTCTTccatCATTAACATGGTGGATGTCTGGAG	NcoI
	0800-MaEXP3proFor	CTATAGGGCGAATTGggtaccCGCACAGCGTCCTCAAGCGG	KpnI
	0800-MaEXP3proRev	TATGTTTTTGGCGTCTTccatTTGCTCCTCTCTCACTCGCG	NcoI
	0800-MaXTH10proFor	CTATAGGGCGAATTGggtaccGCTTGATTGAGGCAATGACAAGGT	KpnI
	0800-MaXTH10proRev	TATGTTTTTGGCGTCTTccatACGAGGATGGTGACGACGC	NcoI
	0800-MaPL3proFor	CTATAGGGCGAATTGggtaccTCGATGACCCTACCACGAGCT	KpnI
	0800-MaPL3proRev	TATGTTTTTGGCGTCTTccatGATGCCATGCCGTAGTATTCAAG	NcoI
	0800-MaPG1proFor	CTATAGGGCGAATTGggtaccGGAGATAAAAATTTGTTAATTTGC	KpnI
	0800-MaPG1proRev	TATGTTTTTGGCGTCTTccatTGTTCCGGACAAAAATAACATATT	NcoI
	0800-MaPME3proFor	CTATAGGGCGAATTGggtaccAGCTCTGTCCGATCCGCTTCG	KpnI
	0800-MaPME3proRev	TATGTTTTTGGCGTCTTccatGTCGTAAAGAGCTCTTCCTCGG	NcoI
	0800-MaACT1proFor	CTATAGGGCGAATTGggtaccTTGAAATCGCCTCAAATTACCAAG	KpnI
	0800-MaACT1proRev	TATGTTTTTGGCGTCTTccatTACCATTGTCACAAACAAGGGGCT	NcoI
