# Supplementary material for: The Banana Transcriptional Repressor MaDEAR1 Negatively Regulates Cell Wall-Modifying Genes Involved in Fruit Ripening
Source: Front Plant Sci. 2016 Jul 11;7:1021. doi: 10.3389/fpls.2016.01021 (PMC4939300; doi:10.3389/fpls.2016.01021)
Supplement: Supplementary file 2 [file Data_Sheet_1.PDF]

Supplementary Sheet 1. Nucleotide sequences of *MaDEAR1*, *MaEXP1/3*, *MaXTH10*, *MaPG1*, *MaPL3* and *MaPME3* promoters. The ethylene-responsive element (ERE), ATTTCAAA, presented in the *MaDEAR1* promoter is underlined. The DRE/CRT *cis*-element [A/(G)CCGAC] in the promoter of *MaEXP1/3*, *MaXTH10*, *MaPG1*, *MaPL3* and *MaPME3* were shown in yellow, translation start site (ATG) was shown in box. The probe nucleotide sequence used in EMSA assay is indicated.

>MaDEAR1 (GSMUA_Achr3T13190_001)-Promoter

TCACTCGACCCATTCCGCACCCGCGCACACGTCGCACTGACTCTGCCTCCTTCCCCTCCCCTCCCCTCCCCGT
CGTAGCCTAAATTACGGGACCTATTCCCCGGTCTCACCCATTTAATGCATCTCAACGACCGTCTCGTACCCATA
TTAACTACGACGCGTCTCTTTATGCTTATTGTTTTATCTTCTTTATTCCTTATTACAAGGATAAATATATACTTTTC
CCTCCACCGCGGTTTCGCGGTAGGCGCGGCTGGCAGATCACCGACCTCATTGTCCTCCGGAAGCGCCGCCC
TTCGCCGGCAGCCGTCAGATCGGGTCCGCAGGTCTCCATCCGACCGTCCGTGATGGTGGCGGCCGCCTGCC
GCGTGTGGTTCCGTGACGAAGGAGGAGACGTCTCCACACGTGCCGCCCAACGACACGTGGCCATTGAAAA
ATGCAGGAAAAATAAAACAATTATT<u>**AATTCAAA**</u>ATAATCCTTTTCAATGCTCGGGGTCACGCACCGCCTAAGA
TTGTCGGCCGGGTAACGTGGCGGCTGCGGCGGTCGACACACGGCCGTCCTATTCCCCGTCTTTTGCGTGCC
ACCCCTTTCCTCTTCTTTCCCCACTATTTTAATATTTCCGTTAATCTTCTTGACGGCCCCGTCACGGATTATGGG
GAAGATGATGACAAGCTGTGGCTTTCCGTCGCACTAACGGCGTCACGCTAGATTGTCGTTTCCTTGATCTTTT
CCGTGTCATTCACTTGGCTACCGGATAAGCAATCCAACAGTGGCGAGAACGACGCGTTCGCTGCCTGCCTCC
ACGCTAAGTATTGCGCGCCCTCCAAATGTGCAGCCAAGTGTTCCTCTGCAGATTCTATCCAGCCCGTTCAATT
TGACTTGATTATGATGGACGGTCGGGATTGGTTTTGAGGCCCCTCCGTAATCTTTCCGACGAGGAGGGGTCT
ATATAGTTTCCATCCAGGGCTTTGGATTGCAATG

>MaEXP1(GSMUA_Achr5G07470_001)-Promoter

CGCACAGCGTCCTCAAGCGGCCGCGTTTCTGCCTGGAGTTGGTCGACGACGACTAAATCTCTCACGGTGGC
GACCACTCGTGGGGACATGCAGAGGAAAAGGTTCGCGAAGGCTATCGTGGACAAAGCACTTCTTCCATGTT
TTTCTAGCTCACAAGTTTGTTGTACTCTGCTCGATCCGTGTTTGCACTTCGCACCGTCTTCATGTGCTTGAACT
CAGCTACATGAACGAGCAAATATTTCAAAGTGCCGGTGAATGATTGGCGTTCTTAATCTGCCGCAAAAGAGT
CAAAGACGGTGGGTAAGTTTGACCCATCATTTCCGACACTGATACTGCCGAGTACACGTACACTATTGCTTGC
GGGATTTGCGGTGTGGCCATTTTCTAGTCGACTCGATGCGTTCGAAGAGCGTCTCTGTCTCTGTCTTCTTCCT
CGCCACGTGAAGAC**GTCGGC**AGCCCGGAACAGCATGTGCTCCTCCTCACATGGTGGGACAGTCCGTATTCA
AAAAGACATTTTTTTCTGACTGTTTTCTTTCACTCGTAGTGATTCATAAACTATAGCCATCTTCCCAGCATACAC
ATTAGTTAAGGACCATAAGGTCTTGCAACGCACACTAATGGTTTAATCTAATCGAACAGAGGAGGTGGATAG
CAGCAACTCATTGTCTAATGATTGCTCATCTTCCATCGTGTAGTGTTGTTTATTAGCGTACTGACATGATTGCG
GCAATTAGTTTGAGCGAGTCATAGTAGCTGGAGCGTAAAGAATGGAGTAATCTCGGCGTGATTGATTGAAG
CACGCATATTTGTGCCGCAGTATGGCACC**GTCGGC**CATACAAGTCCGATTGAGGCCCTTTATTTCTTGAACTG
CTTCGGCGCGCATCTTTGGATTTCCAGCTCCGATACCTATGAACACAGCCTTCAAAGAGTGAGTCAACAATG
TGAACTGCGCAACTTCTTCCTCTGCACGCCATTCACATCATCTTCCTCTGCTTCTGTTCCATCAGCGAGACTTA
TTTGCGCTTCCAACTAATCTTGTCTTGTCCTGCCCTCTCCCCATCTCCTTACCTATTGTCGAATGTAAAGAGAAC
CCCAAGAGAACACAACAGGATAAACGTGGCACCATGAAGTTTCTTCCATGAAAGCAGATGCATTTGGGTGT
GTAGTTGGTTTTGTCACCGGGACGATTGCATGTGCTCCTGATTTGGATGATCGCTGCGGTCGTCTTCAAGCCT
ATGGCTGGCCCATGGCCGCCACCAACTTGTCTCTTTCCAGAGATTGAAGATGAAGAAACCAACTCCAACCCT
CACTCTCAACTCGTATAAAGACACAGCCTTTCCCTCCGCTCCTCTTCACTACTCCTCCTGTTTGCTCACTCACG
ACTCCAATCTTTCTTTAGTGGGAATAGTTCCAATG

MaEXP1-probe for EMSA:

TCTTCCTCGCCACGTGAAGACGTCGGCAGCCCGGAACAGCATGTGCTC

>MaEXP3 (GSMUA_Achr10G26620_001)-Promoter

CGCACAGCGTCCTCAAGCGGCCGCATTTGTGCCCCCTCGACTTGCGTCTCTCCCATCTACCCTCTCTCACGAC
TCCCCCTCCTTCCCTACCAGTACTGTTATTGGCCCTTGGCTTAACCCCACCCACGTCCGTAAAATTAATCCAAT
CACAAGACAGATAGGCCGTGGGCTGACAGTCTCTGGCGTAACGCGACTTATTTCAGTCTCCTTTTCTTATGC
GGTAATAATAATAATAAGTAAAGTTGGATCTTAACGCCG**ACGGAC**ACCGTCGTCCACGTGGAAAGATGGGAC
GAT**GTCGGT**CCCGCTCGAGGTTGACTGGCGCGCAAAATCTGGACCGTCCAATACGCGATGGGCCCACCGCA
AAATCACGAGTTCGGTGGGTGAGATGTGAACCAAAGCGAAAGACTAGCCCAGACGGGTGCTAATTTCCGG
GTAATCGAACGTCGGGTGACTGCTTCTCTCCGCCAAATCCCCGATTTTTGATTCGAAGGAAAATTATTCCCCC
CTATTTTATTTATTTTCTTTTACGAAAATAAAAAGAGATCAAAAAGCGTGGATAAAAGGTCGTCTAATCAGAA
CC**GTCGGT**GGCGGCACAGCTTGCGAGGGAAGCGATCCGAGTACGATTACAGTGCCCGTCCGATTTCCCACT
TCACCGCGACGCAACGTTAGTTACCCGTGGCTCGCCCCGATAAAATCTCAGCCGTTGGTATCTCATCGT**ACCG**
ACCACGTTGCAGACACAGCTATGATGGGAAAGTGGTGCGGTGGAGCCCCCAATCCTTTAATCTTAGCAGAA
AAGACTCTGAGTGCCATCGTTTGATCTCACACTCACACCTCCTCTGCTCGAGCCTACAAATACCCCACGCTTG
CCTCCCTCCACATCCCCATCCTCCAAACCCTTGATCGCGAGTGAGAGAGGAGCAAATG

MaEXP3-probe for EMSA:

CAGCCGTTGGTATCTCATCGTACCGACCACGTTGCAGACACAGCTATG

>MaXTH 10 (GSMUA_Achr10G10260_001)-promoter

GCTTGATTGAGGCAATGACAAGGTCTTTCTCATAATACAAAGTGGTATCATGAGTTCGCAATCTCAATGAGCC
CATCGCTAGAATGTTCAAATGAAGCACAGTTGTTGGTTTCTCGTGTTATCGGTGTTCACTAGATTTCTTTGAG

GACATCCCCTTGTGTCAGATAAGGGAATGACCATGACATGTTCATCTGAGTTAACTTGGTTTTATAGCTGCTC
TTTTTTTTTGAAATTAGTGCTGCACAAACAAAGCTGCAAAGCCGTAGCAGCATGTTTCATATCGTCAAACCAC
AGTTGATCCAACACCCCCTTAGATTGTTTGAATTTCCGTCGCAATTAGAATTCGACATAATGCTAATGCATAAC
CATTCATGAACCCATAAACTAACTTGTGTCTATTCTTATGCAGCTTGAGCAGAGGATCCAAGCATATGGCAGC
GAACAACAAAATTAGAAAAAAAGCGACACACACGATATCATATCATCTGCACTTCGTGTTCCTGTAAATTGTA
CTGTTTGACTTCACACATTGGGTTCAAAACATATCTCTGAGACCAATTGGGAACTTGGTAGGTTAGTGATGGT
AACAAGTCTCATGCATGTCACTGGGAATGTAGAACCTCTTCAATCTTTGTGGAGGACTGACGTTGATAACTG
ATTCAAACTTGATATTAAACATCAATAACTTTAACCCGACTTATTTGTTATCGGGTTGGTCCGATACGACCTGAT
TAACCCCATTATTGTCATTGCTCGTTGTTGATTAAGCTCAATGATGAATGAGACTTGTTGGGTATAAAACAGAT
CAAAATTCAAGAATAATTTGACCTATCAAGGTGTGGGTCATATCGGATCAAATGGGTATCCAAACCCGAGTCT
GATAGATGGTAAACGGGTCAGGCTGATCCATCCATTTGCAATACAAACCCGTCATGTTGTCANNNNNNNNNN
NNNNNNNNNNNACCGTTACCCGTACTATATATATATATATATATATATATAAGGCTTCCGACAAA**GCCGAC**TACTC
AACCGAACCGATCCGAAGAGTCCCGTGATG

MaXTH10-Probe for EMSA:

TATAAGGCTTCCGACAAAGCCGACTACTCAACCGAACCGATCCGAAGAGTCCCGTG

>MaPL3(GSMUA_Achr7G15620_001)-promoter

ATCGATGACCCTACCACGAGCTTAGATATTGCATCATAAAGCAGGTTGGTTGGGGGGAGAGAGAGAAAGAG
ATCGATCGTAGGGACAGTGGCTGAAAAAGAACATGGGGGTATTTATTGGTCCAAGTACACGGACAGGATTA
GGGTTCAAAGGATGATGATAGGAGAGGACTCTCGGTCAACACCCCCACGTCGAAGCAGACATCCGTGCATG
GGCTTTGTCCGCAAACCGC**GTCGGT**TCATGTGATGTGGATCACGTACAGTTTGCGAGGACGAGC**GTCGGC**G
GCCGCTGACACAGCAGCGATGGACGAAACGTGGTGGCGCCGCTTCGTCTCTTCGTCCACGGTAAACGAAG
AAGACCGTCAAATCCTTTTGCATGGGTGGGGAGTCGGACGTGGTTTTATTGCGCTACTGCAGATTGATGTGA
AGACGGATACTGCCAACGCAAATAAACAACTCCAAAGAATATTGCTGAAGTGAAGAAAGCGATCTTTAAAC
AGAGCCTCACGCGGAGACGCATTAAAGTAGTGCAGTACAGCCCTTCTTATGTGGGCGCGATTCTTATTGGCG
TTACCATTTTATCTCACTACTCAATTCTCACCCACTTATATAGAAACATTAATATCATCAGGTTATGAACTCTTTG
TTCTATAGAAAAATGAAGATTAAAGCCATGTCTTTATGGTACAGTAGATTGCACGCAACCAAGTTGCATTAAT
GAATCTCAAAAGCCAAGTGGACATGCCCCAAATTTAACCAAGCTAAATGCAGCTCATACGTGTCGCCGATCC
ACCGCTTATGTACTCAACAAACGACCATGGCTTTGAAAGCTTCGGATAGAAGATTACAAGAACCAATTCAAC
CTCACCACAGCTTTCCACAGTCATAAGAATGAAACTTGACTACAAGTTCCCTACGTTGGTCCAAATCCACCGA
AACTATGTTTTATGTGGGTCTTCTTCTTGATTTCCTTGCTCTTTTGAGCTCAATTAGCAGAAAGCTTCTCGATG
GAGGGCGCGGCAGTTCCAACGCTATCTCTTTTTATCTTTGTGCAGCTGGCACGCGGCTCTGTTTGCTCTATAT
AGCACAATCTGCATGTAGTTGTGAAGCTCATCACCTCTCCATTAATG

MaPL3-probe for EMSA:

CCGCGTCGGTTCATGTGATGTGGATCACGTACAGTTTGCGAGGACGAGCGTCGGCGGCC

>MaPG1(GSMUA_Achr3G13670_001)-promoter

GGAGATAAAAATTTGTTAATTTGCATGATGTAAAACTTGCTAAAAGTGTTATCTTGCAAATCTGAAGAGAAGT
AATGTTTGCTAGCTTACACATTTCAAGAAGATACAAAACATGATATCTTGCACTTCTCAAAAGAGAAGAAAG
AATTCATTAGTTTGCATGTTCTAAAGTGATGAAAAATTTGCTAAATAGTTAGGCTTTCATATCTCTAAAACTTGA
TAGTTGTACTTCTTCTTTTTGTTGATGACAAAGGGGGAGAAGATATGATGATATGAGAATCATACATGGTAGA
ATGTAATATATTTTGATATTTTAATATCATGATGATTTGAAATATTTATGATGATGACAGCCTCAGAGATTGAGTT

TTTCGAGTTTTCCAACTCACAAGCGGTTCCACCGCTTGTTCTGGCGGTGCCACCACTTGACCCAACTTTTGA
ATCACTGAATGAGCCTCCCAATGAGCCCAAATCAGTCTCAATTAAGACCCAATTGGCCCCTAATTGAGTTAGC
ATGATTACACCAAAAGCTAACTCAATTAGCCCCCTAAACTACTTCGATCTTAGGCAAATGATTACAAAGCATG
AATCTTTTGTCCAGCATGTCATTGGTTCATCCGGCGCTCGTCCAATCTTTCGACGCATCATCCTCTCCTTAGGC
ATATTACCCAATCGGCATGTTGACTCCCGTAACTTTCGATCTCCTTAGTGCAATGTCCGATACTTCGGCCCAAT
GCCCGAATTCACGGCATAAAGCCTTCTGCGACACGTCGATCGATCCTTCGGCTCGACGTCCAATCTTCTAATA
TGTTCACTCCGGCCCAATGTCCGATTCCTCCTGCTTTAATCAATTTGTCTCTACTTGATCGAAGCTAGTTCTGC
GTCACTCAAAACACAGATTAGATCACAAACTTATTAACTAATTTCATCATCAAAATTCGAGATTCAACAGTAAT
AACATTCCTTATCCTTGTGACACCATCTATATGAATCTCAAAATATTTATAAATCTTTCTTGTGGACTTATTCTCAT
GGCAATATTTCTTATCCCGGTGACAATAGTTTTTGAAATTTTAAAATGCTCACTAGAAATGAGACAACGCTTG
TGCAAGTGTTTGATTTTGCTCTTTCTTATTAAGACCTTTTTTTTTACTTAAAATAAAAATAAAGAGATTTACTCG
ACGTAGAAGATGAGAACAATAGTTTCAAATCGAGTGCTGCCACGTGTAATAAAAGAGCTATCCCGCAACAAC
GTTGCAGATGC**ACCGAC**CTCATTCAATTCGTGGGGAAGAAGAGGACACGAGAGGAGGAGCAGAGACCGG
TCATCCTCAACTTAATCGAGCGTCATCGTGATGGGCCGAATCAAGCGGCGAATCAATCGTTTTCCACGCGGC
GGTGCACTTGCGTCCGATGCTCCTCATCTATCATCCGTCCGTCGATCCAAGCACCGAGTACGGATCCCATCCC
CTCCCCATCTGCGCAACTTCGTCGACGGAACTGCCCATATTGATGGGTCTCTTCCGCCCGATGGAGTACCTGC
CTGAGCTAATTTACAGACAACGGATGTTCTTTCCTCCCTTGGCTCGCTACCCCTCACAACCGCAGAAATCCCA
TCCCGACCCGAAGTTGTGGTGGTGGTGGCCATGGTGGATGATCAATATGTTATTTTTGTCCGGAACA

MaPG1-Probe for EMSA:

CCCGCAACAACGTTGCAGATGCACCGACCTCATTCAATTCGTGGGGAAGA

>MaPME3 (GSMUA_Achr11G20560_001)-promoter

TAGCTCTGTCCGATCCGCTTCGGACGGTGAGCTGACAAGTCATCACCAGCTACACACCTTTCCTTCTTCTTCT
TCTTCTTCT**GCCGAC**CTCGTCCCACCTCCGAACTGCACATCTTGACTCCATTGTGGAAGTGTCCCAACTCTGC
CTGTCTTCATGCAGAAAATAGCAGCTTATGTATTCCCTTCGTTGGTCTCTTCGTCTCTTGCTTATTAGTCTTGGA
ATACATGTGTGTGACAGCCTGATGCATTATGATTGCAAAACCGTGTTGCGATGCATCTTTTCCAGCTTCCAGTA
GATAACATCAAAATGTACCTTTGTATTTTTCTTCATGTTCTCAATATAATATTTATTATTTTATTGAACATCTGACA
TAGGTTTAATTAGAATCATAATTGACTTCGATCGTAATCAGTTTGACCGATTCAATCTCAGTTCGGCAATTAGC
CGAACCGGTACGACGGATTGGGTTCGAAAAAGTTGGTCCCAAACCACGAAGACTACGAGGATACAGCTGC
GGTGGTCAAAAGATGTATGTTAATTGTCTCCTCTCCTCGATTCTCGCTATGACTTGTGTTCTTACGTTTCTTCAA
TGTCAAAACTACAGAGAAACCACATCTAAGATTGAGCTTTTTGTCATCGGTCCAGATTTGTGTACTTTACTCG
GCAAAAGCTGATTCACCCTTCTTAATCGTTAGTCATTCTATTTCCTTCTCTATAAACATAAACATGTGTACTCTCT
CTCTCTCTCTCACTTCCTACTCGACGCGTTTCATTGGTCTCGAAAGTCAACAATGTTGACCTAATTAGTCTGAT
ATATCTCATATGTTATGCTGATCGGTGTAGTAGTTTGCAGCGTGGTTTGACTTCCTCAAGAATGCGAAGAACC
ATTTCCCTTGGAAGGAGTGCGAGAAATGACCACGAAGACCCTCGAACCCTCCTATATAACTCCCCTGTGACC
CCTCGCCACCTTCGTCGTCCCGAGGAAGAGCTCTTTACGACATG

MaPME3-Probe for EMSA:

TCCTTCTTCTTCTTCTTCTTCTGCCGACCTCGTCCCACCTCCGAACTGCA
